# Concomitant spinal cord, vertebral body, and paraspinal muscle infarction

**DOI:** 10.1002/jgf2.669

**Published:** 2023-12-17

**Authors:** Yoshitaka Tomoda, Satoshi Fujita, Koji Uhara, Yusuke Yasumoto

**Affiliations:** ^1^ Department of General Medicine Itabashi Chuo Medical Center Tokyo Japan

**Keywords:** Neurology, spinal cord infarction, vertebral body infarction

A 76‐year‐old woman presented to the emergency department with a sudden onset of back pain and numbness in her left lower extremity. Her sensory impairment initiated in the dorsal aspect of the foot, progressively radiating to encompass the entire lower limb within a span of 1 day. Her medical history included coronary heart disease. On examination, she had partial muscle weakness in her left lower extremities and hypoalgesia in her left lower extremity, which extended below T6 level, including bilateral lower extremities the following day. A laboratory analysis revealed a normal platelet count and no coagulopathy. Moreover, brain computed tomography (CT) and lumber magnetic resonance imaging (MRI) on Day 2 were unremarkable. However, follow‐up T2‐weighted MRI performed on Day 8 revealed a hyperintense lesion in the spinal cord at T5–T6 without spinal canal stenosis (Figure 1A,B). Additionally, T2‐weighted MRI revealed high‐intensity areas in the right half of the T6 vertebral body, and gadolinium‐enhanced T1‐weighted MRI revealed enhanced lesions in the right latissimus dorsi, which were findings consistent with spinal cord, vertebral body, and muscle infarction (Figure 1B,C). Contrast‐enhanced CT revealed no evidence of aortic dissection or embolism. The patient's symptoms gradually improved after antiplatelet therapy including aspirin and clopidogrel, and she was subsequently transferred to the rehabilitation center.

**FIGURE 1:**
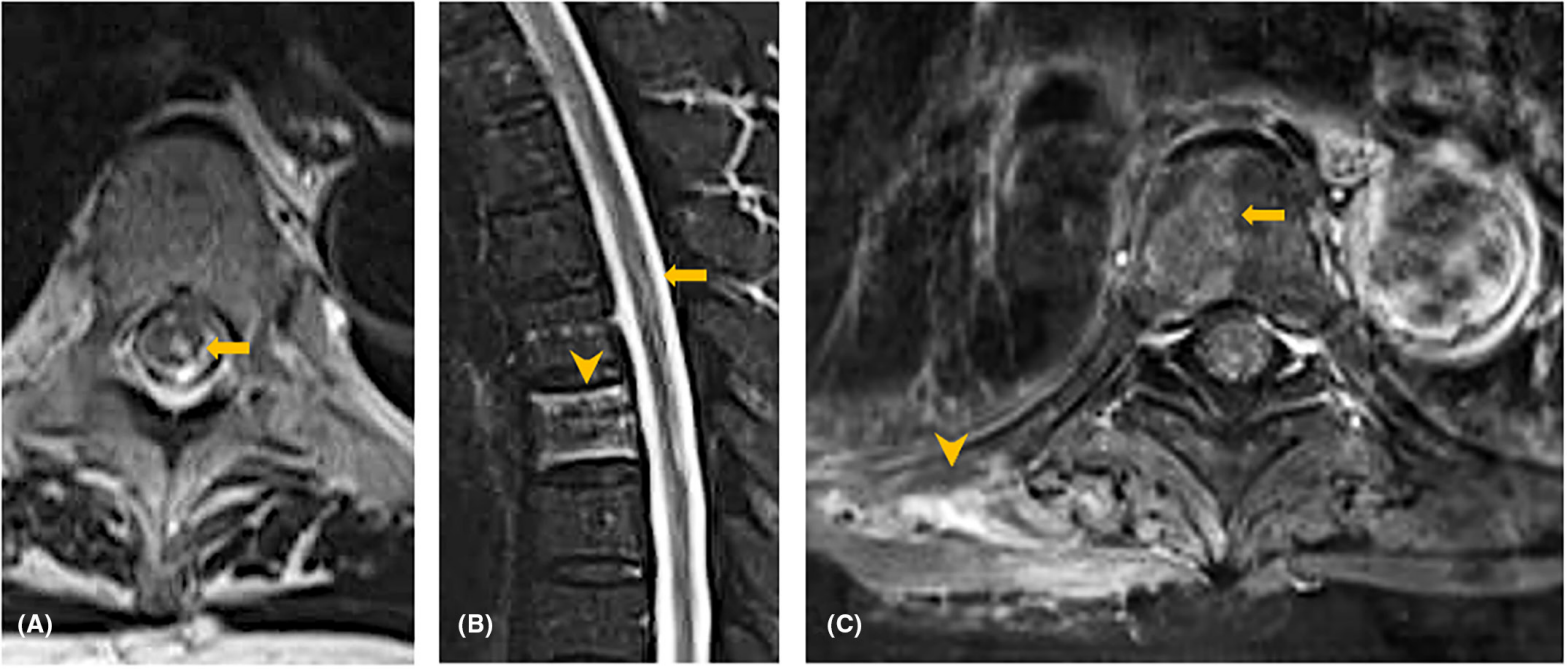
(A) T2‐weighted magnetic resonance imaging (MRI) revealed hyperintensity spot in the spinal cord at T5 level on axial view (arrow). (B) T2‐weighted MRI revealed longitudinal hyperintense lesion in the spinal cord at T5–T6 level (arrow), and hyperintense lesion in the T6 vertebral body (arrowhead) on sagittal view. (C) Gadolinium‐enhanced T1‐weighted MRI revealed high‐intensity areas in the right half of the T6 vertebral body (arrow) expanding to the right latissimus dorsi on axial view (arrowhead).

Spinal cord infarction (SCI) is a rare condition that accounts for 1% of all strokes.^1^ Although the etiology of SCI remains ambiguous, estimates have indicated that approximately 75% of SCI patients exhibit vascular risk factors. Moreover, various conditions, including aortic surgery, atherosclerosis, aortic dissection, cardiogenic embolism, or systemic hypotension, can cause SCI. The clinical features of SCI include rapid neurologic deficits with severe back pain, with an onset‐to‐nadir deficits duration of 12 h. Accurate diagnosis of SCI remains challenging and is based on a combination of clinical symptoms and characteristic MRI findings, including T2‐hyperintense patterns and linear gadolinium enhancement. While MRI has been the most useful tool in the diagnosis of SCI, normal MRI findings are often observed within the first few hours after SCI. Furthermore, there are no specific therapies proven to limit SCI, and treatment for underlying causes is usually suggested. Mortality rates after SCI depend on the level or severity of ischemia or underlying etiology.

Thus, considering SCI, concomitant vertebral body infarction is uncommon, with reports showing a prevalence of 4%–8%.^2^ Similarly, only a few cases of concomitant paraspinal muscle and vertebral infarctions have been reported.^3^ However, the simultaneous presence of vertebral and paraspinal muscle infarction offers valuable information regarding vascular involvement and can help rule out transverse myelitis, which is a differential diagnosis for SCI.^4^ The radiculo‐medullary artery, which supplies to the spinal cord, originates from the segmental arteries branching from the aorta. Conversely, the ventrolateral parts of the vertebrae and paraspinal muscles are supplied by the anterior spinal artery and posterior spinal branch, respectively, both of which also originate from the segmental artery. This indicates that SCI with vertebral and paraspinal muscle infarction should have occlusion proximal to the segmental artery or aortic pathology. Therefore, clinician must consider the characteristics of SCI and should have a high index of suspicion in cases of acute myelopathy with vertebral and muscular infarction.

## CONFLICT OF INTEREST STATEMENT

The authors declare no conflict of interest for this article.

## ETHICS STATEMENT

None.

## PATIENT CONSENT STATEMENT

Written informed consent was obtained from the patient for the publication of this case report.
